# Meetings of Societies

**Published:** 1902-06

**Authors:** 


### MEETINGS OF SOCIETIES.

### Bristol Medico-Chirurgical Society.

*March 12th, 1902.*

Dr. Barclay J. Baron, President, in the Chair.

This meeting was devoted to a special address and discussion on the **diagnosis of oriental or bubonic plague,** by Dr. Klein, F.R.S. The President introdnced Dr. Klein. Numerous photomicrographs were shown with the aid of a lime-light lantern and screen. Drs. R. Farrar, D. S. Davies, J. O. Symes, and Hancocke Wathen discussed the address. (For the full report of the remarks of Drs. Klein, Farrar, and Davies, see p. 108, *et seq.*, of this *Journal.*)

*April 19th, 1902.*

Dr. Barclay J. Baron, President, in the Chair.

The President spoke of the great loss the Society had recently sustained through the death of three of its members —Dr. E. Long Fox, Dr. Bonville Fox, Mr. R. H. Chilton,— and it was resolved that the sympathy of the Society with the relatives of the deceased should be expressed through the secretary.

Dr. F. H. Edgeworth showed a **case of locomotor ataxy with interstitial keratitis.** The point of greatest interest in the case was the association of a disease, locomotor ataxy, usually secondary to acquired syphilis, with a disease, interstitial keratitis, usually due to congenital syphilis. The speaker had now discovered that the patient, an adult man, had been shown to the Society five or six years ago by Dr. Michell Clarke as a case of tabes secondary to congenital syphilis.—Dr. Michell Clarke stated that about the time he had shown him the man suffered from severe gastric crises, and perhaps from intestinal crises as well as from many other symptoms of tabes.— Dr. H. E. Stack asked if Dr. Edgeworth had seen any other cases of interstitial keratitis appearing so late in life.—Dr. Edgeworth replied that he had seen one other case.

Mr. H. Elwin Harris showed a **patient with almost complete adhesion of the soft palate to the posterior wall of the pharynx.** The patient was a boy of twelve who had had severe ulceration of the throat two years previously. The soft palate was so completely and smoothly united to the posterior pharyngeal wall that the line of junction was difficult to detect. A fibrous whitish streak represented the uvula. A small hole through the soft palate alone effected a communication between the mouth and naso-pharynx. Fluids did not regurgitate through this hole, and the patient retained the power to blow through the nose. Mr. Harris was not in favour of attempting any operative relief for the condition.—Dr. Watson Williams and the President also approved of non-intervention.—Dr. Shingleton Smith alluded to a case he had seen, not so advanced perhaps as this, in which dilatation of the space between the palate and the pharyngeal wall had been employed with success.

Mr. T. Carwardine showed a **patient with primary syphilitic sore on the leg.** The patient was a female child. A copious rash had developed after the patient had been a short time under observation.—Mr. C. A. Morton discussed the diagnosis of chancre as met with on the lips and fingers. He had seen a chancre of the lip which bore a resemblance to epithelioma. There had been hard glands at the angles of the jaw in this case. Two cases of chancre on the finger he had seen resembled sarcomatous growths.—Mr. Munro Smith showed two drawings of chancres on the fingers, and alluded to the interesting question as to the amount of induration which was to be expected in such cases.—Mr. Mole mentioned a case in which he had seen a vulval sore removed by operation with the diagnosis of epithelioma. Subsequently a syphilitic rash developed, showing the true nature of the sore.—Dr. Watson Williams alluded to the value in diagnosis of the bubo which developed quickly in these cases, as contrasted with its slower development in malignant disease.—Mr. Elwin Harris related a case of chancre on the fingers in a medical man, the development of which he had watched. No induration had been noticeable.—Mr. Carwardine considered that the diagnostic difficulties were greatest when the conjunctiva or the external os of the uterine cervix were affected. Many true chancres were without induration. Chancres of the vagina and vulva were frequently without induration.

Dr. W. H. C. Newnham showed a **specimen of ruptured tubal pregnancy,** in which the rupture had occurred fourteen years before the tube had been removed. The patient, a woman aged 42, had been invalided with chronic pelvic pain. Abdominal examination revealed nothing, but on vaginal examination a very hard swelling was felt lying behind and to the left of the uterus. It was the size of an orange. At the operation this hard mass and the adjacent left ovary and Fallopian tube were removed. The mass proved to be a fœtus. The patient gave a history of having had a miscarriage fourteen years previously. She had been ill for twelve months after with obscure abdominal symptoms. Examination of the specimen showed that the portion of the tube removed was healthy for a certain distance from the uterine end, where there was a cicatrix, and beyond that it was healthy again. The speaker believed, therefore, that the illness fourteen years previously had been really due to the rupture of a tubal gestation.—Dr. Walter Swayne agreed with the interpretation given of the case. Considering the proximity of the rectum, bladder, etc., it was surprising that an abscess had not ensued. There were undoubted cases of calcification of fœtuses arising from tubal gestation. He knew of one such lithopædion which was almost as large as a full-term fœtus.

Dr. Fisher showed a **specimen of a tuberculous tumour of the brain, which had become quiescent and calcareous**, and read a paper on "The Healing of Tuberculous Lesions of the Brain." He read brief notes of reported cases in which similar calcareous masses to that shown had been found at autopsies, and referred to others where the symptoms of cerebral tumour had disappeared and the patients had recovered with loss of sight. It could not be proved, however, that in these cases a tuberculous tumour had been present, since symptoms undistinguishable from those of a cerebral tumour could be produced by chronic internal serous meningitis. Dr. Fisher had seen such a case, and others had been recorded by Diller and Oppenheim. Symptoms of cerebral tumour could also be produced by thrombosis of cerebral veins, and in a case recorded by Englehardt, in which a cerebral tumour had been thought to exist, nothing was found at the autopsy but atrophy of the optic nerves, the sequence of optic neuritis. The question of the possibility of recovery from tuberculous meningitis was then briefly dealt with. It was pointed out that only within comparatively recent years had simple posterior basic meningitis been widely recognised, and that most cases of supposed recovery from tuberculous meningitis were probably examples of this disease. There was, however, some evidence to support the view that recovery from tuberculosis of the meninges might take place, and brief reference was made to records of cases by Rilliet, Rotch, Jansens, Carrington, and others.—Dr. Stack alluded to some specimens he had seen demonstrated at the Ormond Street Hospital. One, obtained from a child which died of tubercular meningitis, showed the existence of five or six old healed tubercular masses, one of them being deep in the brain and the others in the dura mater. He asked if it was certain that the specimen shown was one of tubercular disease and not one of syphilis. In its superficial position, it resembled the latter.—Dr. Michell Clarke said that three points of interest had been raised by the paper under discussion—viz., the question of the recovery from tubercular tumours; that of the nature of those cases which gave the clinical signs of intra-cranial tumour, and yet recovered; and that of the possibility of recovery from tubercular meningitis. He quoted Williamson's case, in which a patient died about two years after recovering from tumour symptoms, and at the autopsy the old lesion was found in a calcareous condition. Dr. Bristowe used to teach that tubercular meningitis might recover, but possibly the cases which had given him that impression had been cases of basic meningitis.—Dr. Newman Neild mentioned a case of spinal meningitis in which calcareous nodules had been found in the meninges. It was possible that small numbers of tubercles might exist in the meninges without exciting the symptoms of meningitis.—Mr. Lace described the case of a child he had been asked to see, in which the cardinal symptoms of cerebral tumour were present. A physician considered the localising symptoms were not definite enough to justify operation. The speaker had advised operation, and so also had an oculist. No operation was done, and the child recovered, but not before blindness had resulted from post-neuritic atrophy.

Dr. W. H. C. Newnham read a paper on a **case of sloughing fibroid successfully removed by abdominal hysterectomy.** The patient, an extremely anæmic woman, came first under his care with a large black mass projecting from the vagina. The uterus reached up to the level of the umbilicus. He attempted to remove this mass *per vaginam*, but only succeeded in cutting off the vaginal portion. The patient subsequently left the hospital for a time, but the tumour grew again, and she was re-admitted and the above operation was performed in spite of the danger involved in attacking so foul and septic a tumour by the abdominal route. No drainage was employed.—Dr. Walter Swayne pointed out how difficult it was to purify a uterus which had reached an advanced septic condition as a preliminary to its removal by an intraperitoneal operation. He had scraped a carcinomatous uterus on one occasion for this purpose and thought he had succeeded in disinfecting the organ, but on examination after its removal he had found that the septic material had been by no means effectually removed.

Drs. Michell Clarke and Newman Neild read notes on a **case of tuberculous pericarditis, and showed specimens.** The patient, a man of 50, had cystitis twelve years ago, the attack lasting about a fortnight. Twelve months before admission to the General Hospital he had an attack of hæmoptysis lasting three weeks. The present illness began six weeks before admission, with pains in the abdomen and vertigo, followed by general soreness all over the body. Then the feet and lower limbs began to swell. On admission, a large pericardial effusion was found, with signs of infiltration of the apices of the lungs with tubercle. The urine contained a trace of albumin, with a light deposit containing granular and epithelial casts, squamous epithelium, and pus cells. The pulse rate varied between 96 and 120, whilst the respiration remained uniformly about 40. The average temperature was 99° F., with variations of one degree. The patient died suddenly. At the *post-mortem* 32 oz. of brown oily fluid were measured from the pericardium. S.G. 1025, alkaline, spectroscopically showing bands of met- and oxyhæmoglobin. A few leucocytes were found to contain tubercle bacilli, chiefly large hyalin leucocytes with reniform nuclei. The heart was dilated and somewhat hypertrophied, and was covered with a thick layer of shaggy lymph separated from the muscle by a firm layer of yellow material in which it was just possible to see minute tubercles. There were also two raised hemorrhagic patches on the left ventricle. The mode of entry was thought to have been from the retro-sternal glands. Each suprarenal contained a large caseous mass in the medulla; it was noted, however, that there was no increase of pigmentation of the skin. The bladder contained two tuberculous ulcers, and the left kidney was converted into a lobulated sac of greenish fluid with some caseous particles; the right kidney showed a patch of caseating tubercle and was much hypertrophied.—Dr. NEWMAN NEILD observed that whilst tubercles in the pericardium were not infrequent, especially in children, yet a definite tuberculous pericarditis was comparatively rare. —Dr. MICHELL CLARKE, remarking on the clinical features of the case, said that the aspect and attitude of the patient were characteristic of a large pericardial effusion; the sharp delimitation of the area of dulness, and the extreme character of the dulness, were noticeable. Amongst the subsidiary signs it may be mentioned that the dull area over the left base, extending across the spine to the right side, was well marked, that there was no bronchial breathing just outside the right nipple (Ewart's sign), and, lastly, that the angle formed by the right edge of the dulness with the upper line of liver dulness was an obtuse one.—Dr. T. FISHER stated that tubercular pericarditis did not cause enlargement of the heart. In pericarditis from rheumatism, almost without exception the heart became enlarged.

J. LACY FIRTH,
*Reporter.*

J. PAUL BUSH,
*Hon. Sec.*

